# Lipophilic Compound-Mediated Gene Expression and Implication for Intervention in Reactive Oxygen Species (ROS)-Related Diseases: Mini-review

**DOI:** 10.3390/nu2070725

**Published:** 2010-07-07

**Authors:** Yukiko K. Nakamura, Stanley T. Omaye

**Affiliations:** Department of Nutrition, University of Nevada, Reno 1664 N, Nevada 89557-0208,USA; Email: yukiko@cabnr.unr.edu

**Keywords:** antioxidant, reactive oxygen species (ROS), atherosclerosis, conjugated linoleic acid (CLA), vitamin E (or α-tocopherol), peroxisome proliferator-activated receptor gamma (PPARγ), nuclear factor kappa B (NF-κB)

## Abstract

In addition to exhibiting antioxidant properties, conjugated linoleic acid (CLA) and vitamin E may modulate gene expression of endogenous antioxidant enzymes. Depending on cellular microenvironments, such modulation reflects either antioxidant or prooxidant outcomes. Although epidemiological/experimental studies have indicated that CLA and vitamin E have health promoting properties, recent findings from clinical trials have been inconclusive. Discrepancies between the results found from prospective studies and recent clinical trials might be attributed to concentration-dependent cellular microenvironment alterations. We give a perspective of possible molecular mechanisms of actions of these lipophilic compounds and their implications for interventions of reactive oxygen species (ROS)-related diseases.

## 1. Antioxidants and Implications of their Interventions in Reactive Oxygen Species- (ROS) Related Diseases

Oxygen is essential for all aerobic organisms to survive and produce energy. During the utilization of oxygen, reactive oxygen species (ROS) are generated as intermediate compounds during metabolic processes, host defense, and exposure to environmental chemicals (e.g., pollutants and toxicants). ROS can cause oxidative damage of other biomolecules, such as carbohydrates and proteins, under conditions where there is a significant imbalance between oxidants and antioxidants. In addition to such adverse effects, ROS can exert beneficial effects, including the respiratory burst of white blood cells as a host defense. When the organism is subjected to mild oxidative stress, endogenous antioxidant defense systems can be upregulated to protect cells [1]. Excess of ROS production is implicated in pathologies of inflammatory and/or chronic diseases, such as cardiovascular disease (CVD), cancer, and diabetes [1,2,3]. Therefore, antioxidants likely play an important role in the prevention of ROS-related diseases. The antioxidant defense system consists of endogenous enzymes, including superoxide dismutase (SOD), catalase, glutathione peroxidase (GPx), and endogenous/exogenous compounds, including glutathione, vitamins A, C, and E. Together, endogenous and exogenous antioxidants work in concert as coantioxidants exerting synergistic effects to counter prooxidant situations [1]. 

Although epidemiological and experimental studies [4,5,6] have indicated that vitamin E and other antioxidant vitamins have health promoting properties, such as anti-carcinogenic and anti-atherogenic effects, findings from human intervention studies have been inconclusive [7,8,9,10,11]. Thus, it is important to reexamine the methodology and outcomes of all the research for possible mechanistic reasons to explain such diverse findings [12]. Perhaps considering confounding factors (e.g., diets and lifestyles) and cellular micro-environmental factors (e.g., dose/concentrations of nutrients/compounds, interactions between nutrients/compounds, gene polymorphisms related to nutrients/compounds, and interactions between genes and nutrients/compounds), we may be able to uncover reasons for such discrepancies [13,14]. For example, researchers might consider studying the actual uptake or blood levels of fat soluble and water soluble antioxidants as correlates to benefit for more accurate interpretation of that nutrient’s efficacy [1]. In this article two lipophilic compounds, conjugated linoleic acid (CLA) and vitamin E (α-tocopherol), and the potential interventions of such compounds are discussed in relation to the antioxidant defense system and atherosclerosis prevention. 

## 2. Conjugated Linoleic Acid (CLA)

**Figure 1:**
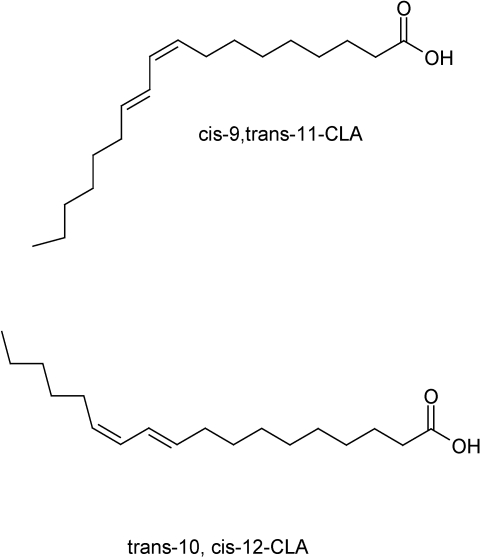
Structures of predominant conjugated linoleic acid isomers.

Conjugated linoleic acid is a group of polyunsaturated fatty acids (PUFA) containing conjugated double bonds in its structures (Figure 1). There are twenty-eight different CLA isomers identified [15]. Most of CLA studies have primarily focused on predominant CLA isomers, the *cis*-9, *trans*-11 and *trans*-10, *cis*-12 CLA isomers. CLA isomers are unusual zoochemicals (or non-phytochemicals), which exhibit health promoting properties, such as antioxidant, anti-carcinogenic, anti-obese, anti-inflammatory, and anti-atherogenic effects, mainly in animal and cell culture models [16,17,18,19,20,21,22,23,24,25,26,27]. Ruminant-derived foods (e.g., beef, milk, cheese) are main sources of CLA isomers. CLA isomers are biosynthesized in ruminant rumen by gram-negative bacteria, *Butyrivibriofibrisolvens*, and exist as unstable intermediates in biohydrogenation processes of linoleic acid to stearic acid [28]. Commercial CLA isomers are produced by alkali-isomerization of linoleic acid, and they include *cis*, *cis*, and *trans*, *trans* isomers at minor amounts [22]. Higher oxidative susceptibility of CLA isomers was observed due to the presence of conjugated bonds, compared to non-conjugated bonds of linoleic acid [29]. Biophysical and biochemical properties of CLA isomers, such as their affinity to membrane cholesterol and impact of cellular membrane permeability, may influence their biological effects, which in turn may be isomer-specific [30]. It is interesting to note that CLA has been available as an FDA-approved weight loss supplement in the U.S. market since 2008. The promise of CLA as a weight loss supplement has been supported by two meta-analyses of Whigham *etal*. [31,32], indicating modest anti-obese effects of CLA isomers, including a decrease in body fat and increase in fat-free mass in humans. Regarding the potential usefulness in atherosclerosis, Kritchevsky *etal*. [33] originally reported inhibition and regression of atherosclerosis in rabbit models fed CLA isomer mixtures for 90 days. However, clinical trials to examine CLA’s anti-atherogenic effects have been inconclusive [34,35,36,37,38,39,40]. 

Although earlier studies suggested that CLA isomers’ anti-carcinogenic and anti-atherogenic effects were mediated through antioxidant mechanisms, recent investigations (see below) indicate that CLA isomers may be involved in regulation of genes, whose products influence ROS generation, such as antioxidant enzymes, through redox-sensitive transcription factors [41]. 

## 3. Vitamin E

**Figure 2:**
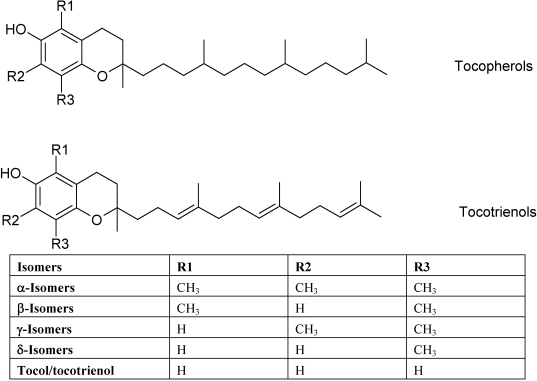
Structures of vitamin E isomers.

For the last decade, the plant-derived lipophilic compound vitamin E, in particular its predominant isomer α-tocopherol, has been intensively investigated in association with its potential usefulness as an antioxidant in CVD prevention. The free hydroxyl group of vitamin E plays a crucial role in scavenging free radicals and superoxide [42] and in protecting PUFA of cell membranes and low-density lipoproteins (LDL) from oxidation. Isomer specificities of vitamin E have been reported [43,44]. For example, tocotrienols, a group of vitamin E isomers containing three double bonds in their tail (Figure 2), exhibit neuro-protective, anti-cancer, and cholesterol-lowering properties, which are not shown by other tocopherols [43] and are independent of the antioxidant properties for tocopherol [44]. 

Although recent findings in α-tocopherol clinical intervention studies directed at looking for CVD efficacy failed to support earlier observational studies [7,9,10,11,45], it behooves us to carefully look for what might account for such diverse findings. Many American adults do not meet the DRI guidelines of micronutrients even when they use supplements, and effects of the use varies with gender, age, and specific nutrients. For instance, supplementation at high doses is associated with intakes above ULs for vitamin C [46], in spite of recommendation to avoid potential adverse effects, supplementation should not exceed 150% of the RDA [47]. Less is done to assist consumer awareness of following DRI’s, there is a need for balance between the antioxidant vitamins (e.g., vitamins C and E recycling), which in turn likely influences the protective and/or other biological effects [48,49]. 

## 4. Cellular Micro-Environmental Factors Influence Actions of Compound and Implications for Molecular Mechanisms of Action of Cla and Vitamin E

Lipophilic compounds, CLA isomers and α-tocopherol, are possible peroxisome proliferator-activated receptor gamma (PPARγ) activators. CLA isomers have an affinity to PPARγ [22,41] and α-tocopherol has structural similarities to troglitazone, a PPARγ activator [50,51]. In addition, computer-based searches of transcription factor binding sites indicate involvement of two redox-sensitive transcription factors, PPARγ and nuclear factor kappa B (NF-κB), in gene expression of selected antioxidant enzymes, human Cu/Zn SOD and human catalase [41,51,52]. 

Due to its high oxidative susceptibility of the conjugated double bonds, CLA isomers can serve as prooxidants or cytotoxic agents to cancer cells, and inhibit breast cancer growth under tested conditions [53,54]. CLA isomers play a role in inflammation. CLA isomers appear to modulate cyclooxygenase-2 (COX-2), which is a source of ROS, and subsequently prostanoids in certain cells/tissues in an isomer-dependent manner [55,56] possibly through NF-κB pathway [57]. In human umbilical vein endothelial cells (HUVECs), concentration-dependent effects of CLA isomers were observed [41], including increases in DNA binding activities of the transcription factors (PPARγ and NF-κBp50), expression of the antioxidant enzymes (human Cu/Zn SOD and human catalase), and ROS generation seen at low concentrations (5μmol/L). At higher concentrations of CLA isomers (10-100 μmol/L), there were increases in DNA binding activities of the transcription factors and the enzyme expression, but no ROS generation. Thus, CLA isomer-mediated gene expression is likely through the activation of the transcription factors. We speculate that effects of CLA isomers at low and high concentrations are related to prooxidants or pro-inflammatory signals to activate NF-κB p50/p65 and PPARγ activators. In CLA isomer-mediated human Cu/Zn SOD gene expression, both NF-κB p50/p65 and PPARγ pathways may be involved in a concentration-dependent manner. Based on our findings, CLA isomer-mediated catalase gene expression may be regulated through the NF-κB p50/p65 pathway. Recent findings suggest to us that [52] the PPARγ pathway may also be involved in CLA-mediated human catalase gene expression. 

Vitamin E can exhibit pro-oxidant effects under certain conditions [48,58]. Vitamin E is also involved in inflammation. α-Tocopherol post-translationally inhibits COX activity in human endothelial cells in a dose-dependent manner [59]. Additionally, γ-Tocopherol suppresses COX-2 activity and pro-inflammatory eicosanoids and cytokines in cell culture models [60,61]. Moreover, anti-cancer effects of γ-tocotrienol are in part due to inhibition of NF-κB p50/p65 pathway [62,63]. In our study [51], α-tocopherol exhibited concentration-dependent effects, similar to CLA isomers, including increases in DNA binding activities of the transcription factors (PPARγ and NF-κB p50), expression of the antioxidant enzymes (human Cu/Zn SOD and human catalase), and ROS generation at low concentrations of α-tocopherol (10 μmol/L) in HUVECs. These findings suggest involvement of the transcription factors in α-tocopherol-mediated gene expression. Interestingly, gene expression of human Cu/Zn SOD was suppressed at middle concentrations (25-50 μmol/L) with an increase in DNA binding activity of NF-κB p50 and without an increase in ROS generation. The DNA binding of the NF-κB p50 subunit is stimulated by reducing agents [64], possibly including α-tocopherol. The NF-κB p50/52 homodimer is a competitor of the NF-κB p50/p65 heterodimer for same DNA binding sites, and the homodimer is associated with anti-inflammatory effects [65,66]. Taken all together, our findings suggest involvement of NF-κB p50/p52 homodimer formation (or NF-κB p50/p65 inactivation), rather than NF-κB p50/p65 heterodimer activation, at least within the range of the experimental concentrations tested (25-50 μmol/L). The findings are consistent with three roles for α-tocopherol: a prooxidant or pro-inflammatory signal to activate NF-κB p50/p65 heterodimer, an antioxidant or reducing agent to induce NF-κB p50/p52 homodimer formation, and a PPARγ activator, depending on its concentrations. Therefore, three pathways may be involved in α-tocopherol-mediated human Cu/Zn SOD gene expression: NF-κB p50/p65 heterodimer activation, NF-κB p50/p52 homodimer formation (or NF-κB p50/p65 heterodimer inactivation), and PPARγ activation. α-Tocopherol also has some influence on human catalase gene expression, for which there are at least two pathways: NF-κB p50/p65 heterodimer activation and NF-κB p50/p52 homodimer formation (or NF-κB p50/p65 heterodimer inactivation). Thus, α-tocopherol may act as an exogenous antioxidant itself but also a regulator of endogenous antioxidant enzymes. 

Overall, either CLA isomers or α-tocopherol may regulate gene expression of the antioxidant enzymes, Cu/Zn SOD and catalase, through two redox-sensitive transcription factors, PPARγ and NF-κB, depending on the respective concentrations of each compound. Subsequently, the compounds appear to modulate ROS generation through regulation of the antioxidant enzyme gene expression. Various known and/or unknown compounds, which possess common chemical and physical similarities with either CLA isomers or α-tocopherol, may also be implicated in the regulation of the antioxidant enzyme expression through PPARγ and NF-κB and ROS generation. 

## 5. Conclusions

There is interest in CLA isomers and vitamin E as lipophilic compounds for preventative and therapeutic strategies of ROS-related diseases. However, whether such compounds exert beneficial or adverse effects are likely to depend upon cellular micro-environment factors, such as: doses/concentrations of compounds/nutrients, interactions between compounds/nutrients and/or between compounds/nutrients and genes, and polymorphisms in genes related to compounds/nutrients [14]. Within cellular microenvironments, a single compound can possess opposite or biphasic effects, *i.e.*, antioxidant/anti-inflammatory *vs.* prooxidant/pro-inflammatory or hormesis. Hormesis is the phenomenon whereby a substance has been found to have beneficial effects at one concentration in contrast to very different effects at higher (and lower) concentrations, such as detrimental response. The phenomenon is widely documented in pharmacology, toxicology, and biology [67,68] and more recently in nutrition [69], for example deficiencies and toxicities of vitamins. In addition, hormesis has also been implicated in nutraceutical research dealing with bioactive compounds that modulate gene expression at specific concentrations [47], as noted above for concentration-dependent different properties of CLA and vitamin E [41,51]. 

Interactions between compounds/nutrients, including antioxidants, are expected and do occur in many biological processes. Outcomes of such interactions include synergistic protective actions of antioxidants [48,49], as well as actions of antagonists/inhibitors. We cannot assume that the outcome of their total actions is equal to the sum of each compound, because cellular microenvironments may lead to less or more overall oxidation rather than some synergistic response. 

Balance, moderation, and variety are the pillars of healthy diet recommendations [70]. Use or over-use of individual dietary components, *i.e.*, supplementation of a single or even multiple compound(s), is contrary to that recommendation and negates the healthy diet concept. Another consideration is that lipophillic compounds have been identified to play a role in gene regulation [23,51,71,72,73,74,75,76,77]. Thus, superimposed on nutrient gene regulation is the role of polymorphisms of genes and individual susceptibility and/or response to intakes of various compounds/nutrients [78,79,80,81]. 
